# Antimicrobial susceptibility surveillance and antimicrobial resistance in *Neisseria gonorrhoeae* in Africa from 2001 to 2020: A mini-review

**DOI:** 10.3389/fmicb.2023.1148817

**Published:** 2023-04-06

**Authors:** Francis Kakooza, Reuben Kiggundu, Gerald Mboowa, Patrick David Kateete, Olga Tendo Nsangi, Jupiter Marina Kabahita, Bernard Ssentalo Bagaya, Daniel Golparian, Magnus Unemo

**Affiliations:** ^1^Infectious Diseases Institute, Makerere University College of Health Sciences, Kampala, Uganda; ^2^Department of Immunology and Molecular Biology, Makerere University College of Health Sciences, Kampala, Uganda; ^3^USAID Medicines, Technologies, and Pharmaceutical Services (MTaPS) Program, Management Sciences for Health, Kampala, Uganda; ^4^WHO Collaborating Centre for Gonorrhoea and Other STIs, Department of Laboratory Medicine, Microbiology, Örebro University, Örebro, Sweden; ^5^Institute for Global Health, University College London (UCL), London, United Kingdom

**Keywords:** *Neisseria gonorrhoeae*, antimicrobial susceptibility surveillance, antimicrobial resistance, Africa, WHO Gonococcal Antimicrobial Surveillance Program (GASP), WHO enhanced GASP (EGASP), ceftriaxone, azithromycin

## Abstract

Antimicrobial resistance (AMR) in *Neisseria gonorrhoeae* (NG), compromising gonorrhea treatment, is a global public health concern. Improved, quality-assured NG AMR monitoring at the global level is essential. This mini-review examined NG AMR susceptibility surveillance and AMR data from the African continent from 2001 to 2020. Eligible peer-reviewed publications (n = 30) containing NG AMR data for antimicrobials currently recommended for gonorrhea treatment were included. Overall, very limited NG surveillance and AMR data was available. Furthermore, the NG AMR surveillance studies varied greatly regarding surveillance protocols (e.g., populations and samples tested, sample size, antimicrobials examined), methodologies (e.g., antimicrobial susceptibility testing method [agar dilution, minimum inhibitory concentration (MIC) gradient strip test, disc diffusion test] and interpretative criteria), and quality assurance (internal quality controls, external quality assessments [EQA], and verification of AMR detected). Moreover, most studies examined a suboptimal number of NG isolates, i.e., less than the WHO Global Gonococcal Antimicrobial Surveillance Program (GASP) and WHO Enhanced GASP (EGASP) recommendations of ≥100 isolates per setting and year. The notable inter-study variability and frequently small sample sizes make appropriate inter-study and inter-country comparisons of AMR data difficult. In conclusion, it is imperative to establish an enhanced, standardized and quality-assured NG AMR surveillance, ideally including patient metadata and genome sequencing as in WHO EGASP, in Africa, the region with the highest gonorrhea incidence globally. This will enable the monitoring of AMR trends, detection of emerging AMR, and timely refinements of national and international gonorrhea treatment guidelines. To achieve this aim, national and international leadership, political and financial commitments are imperative.

## Introduction

Antimicrobial-resistant (AMR) infections are a threat to the global public health and associated with significant morbidity and mortality (World Health Organization, 2014; Price, 2016; Bloom et al., 2017; Tayler et al., 2019). People living in resource-limited settings like Africa are disproportionately affected by AMR infections (Toner et al., 2015; O’Neill, 2016).

Sexually transmitted infections (STIs) are public health concerns worldwide and the World Health Organization (WHO) estimated in 2020 that 82 million global incident gonorrhea cases among adults occur annually, with the highest incidence in Sub-Saharan countries of the WHO African region (Rowley et al., 2019; Unemo et al., 2019b). Complications and sequelae of gonorrhea disproportionally affect women and include pelvic inflammatory disease, ectopic pregnancy, infertility, and increased HIV transmission and acquisition (Walker and Sweet, 2011), and Sub-Saharan Africa is the region most affected by HIV globally (UNAIDS, 2022). Antimicrobial therapy is the mainstay for management and control of gonorrhea. However, AMR in *Neisseria gonorrhoeae* (NG) has emerged to all antimicrobials available for empirical first-line treatment (Unemo and Shafer, 2014; Unemo et al., 2019a,b, 2021).

The WHO has listed key AMR priority pathogens, which included NG as an urgent public health threat for which the global AMR surveillance needs to be substantially enhanced (Seale et al., 2017). For these priority pathogens, the WHO Global Antimicrobial Resistance and Use Surveillance System (GLASS) enables countries to generate quality-assured AMR data to inform national and international treatment guidelines, public health policy and action (Wi et al., 2017; Unemo et al., 2021). For global NG AMR surveillance, the WHO GLASS has liaised with the WHO Global Gonococcal Antimicrobial Surveillance Program (GASP) and WHO Enhanced Gonococcal Antimicrobial Surveillance Programme (EGASP). The WHO GASP includes all WHO regions and it was in 2012 further supported by the WHO global action plan to control the spread and impact of AMR in NG (World Health Organization, 2012; Wi et al., 2017). This emphasized enhanced regular, quality-assured, and comparable global NG AMR surveillance data (World Health Organization, 2012; Wi et al., 2017; Unemo et al., 2019a). Unfortunately, in African countries, which also have the highest incidences of gonorrhea (Rowley et al., 2019; Unemo et al., 2019b), the surveillance of etiologically diagnosed gonorrhea and NG AMR has been exceedingly limited (World Health Organization, 2012, 2021; Unemo et al., 2021). Accordingly, the true burden of gonorrhea and NG AMR is basically unknown in most African countries. For example, in the latest WHO GASP publication including global NG AMR data from 2017 to 2018, only 5 (10.6%) of the 47 WHO African countries provided AMR data for a total of only around 700 NG isolates (Unemo et al., 2021). Furthermore, standardized and representative molecular surveillance of resistance to currently recommended gonorrhea treatments such as ceftriaxone, cefixime and azithromycin has been mainly absent in Africa, and it is imperative to substantially enhance this surveillance, ideally using genome sequencing, in Africa as well as worldwide (Donà et al., 2017; Golparian and Unemo, 2022). The very large scarcity of phenotypic and molecular NG and AMR data makes evidence-based refinements of treatment recommendations for gonorrhea, as well as establishment of appropriate syndromic management guidelines in Africa, very difficult. A substantially enhanced, standardized and quality-assured NG AMR surveillance in Africa is critical toward achieving the WHO health-related global development goals, specifically Sustainable Development Goal 3 given the impact of STIs on reproductive health, HIV transmission (which remains very high in many African countries), and the risk of global transmission of multidrug-resistant and extensively drug-resistant NG (Unemo et al., 2019a, 2021).

In this mini-review, we summarize NG AMR surveillance, AMR testing methods, including internal quality control strains, and AMR data from the African continent from 2001 to 2020. We included papers examining one or several of the four WHO GASP, EGASP and GLASS recommended antimicrobials, i.e., ceftriaxone, cefixime, azithromycin and ciprofloxacin. These antimicrobials represent current or recent first-line treatment for gonorrhea in most countries worldwide (Unemo et al., 2021; World Health Organization, 2021).

## Methodology

**Figure 1:**
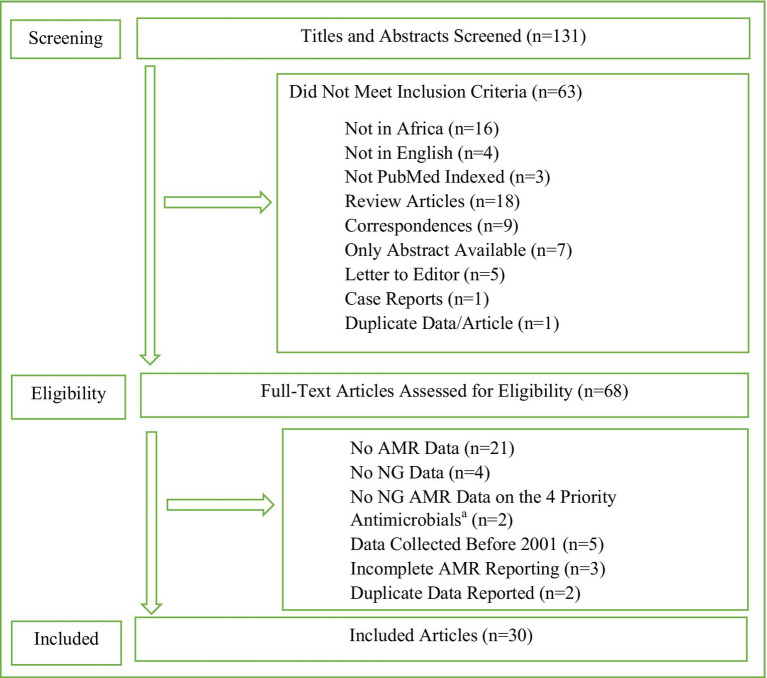
Search terms and strategies, and data extraction template. Search History Sorted by Best Match 2001 to 2020. *Search String:* (Gonorrhea OR Neisseria gonorrhoeae Infection OR Infection, Neisseria gonorrhoeae OR Infections, Neisseria gonorrhoeae OR Neisseria gonorrhoeae Infections OR Gonorrhoea OR Gonococcal Infection OR Gonococcus OR Gonorrhoeae OR Gonorrhoeae growth OR Gonorrhoeae Infection OR Gonorrhoeae Infections OR Gonorrhoeae Strains) AND (Antimicrobial resistance OR Antibiotic resistance OR Antibacterial drug resistance OR Antimicrobial drug resistance OR Antimicrobial drug resistances OR Drug resistance OR Drug resistance, bacterial OR Drug resistance, microbial OR Drug resistances, microbial OR Drug resistance, multiple, bacterial) AND (Africa OR Africa South of the Sahara OR South Africa OR Africa, Western OR Africa, Southern OR Africa, Northern OR Africa, Eastern OR Africa, Central). *Results:* 131. AMR, antimicrobial resistance; NG, Neisseria gonorrhoeae. ^a^Ceftriaxone, cefixime, azithromycin, and ciprofloxacin.

Eligible peer-reviewed articles including NG AMR surveillance and AMR data in Africa were identified through an advanced search for best match in PubMed online databases for publications from 2001 to 2020. The search terms and strategies, and eligibility criteria are fully detailed in Figure 1. The extracted variables included year of publication, country, sample size, surveillance period (collection of isolates), methods of AMR testing (agar dilution, minimum inhibitory concentration [MIC] gradient strip test, such as Etest, or disc diffusion tests), antimicrobials tested, interpretative criteria used (e.g., European Committee on Antimicrobial Susceptibility Testing [EUCAST, www.eucast.org] or Clinical Laboratory and Standards Institute [CLSI, www.clsi.org]), and quality control strains.

## Results

### *Neisseria gonorrhoeae* antimicrobial resistance studies in Africa from 2001 to 2020

The advanced search generated 131 articles with 30 articles included according to the selection criteria (Figure 1). The 30 articles and their results are summarized in Table 1 and included results from 13 (24.1%) of the 54 African countries. All 30 eligible studies were published during 2006 to 2020, i.e., there were no eligible studies in 2001–2005, and 25 (83.3%) of them were published during 2010–2020. The sample size highly varied (from 4 to 458 NG isolates), with 56.7% (*n* = 17) of studies examining AMR in ≥100 NG isolates per setting and year, i.e., in accordance to WHO’s recommendations. Study period also widely differed with 40% (*n* = 12) of the studies conducted during 0–6 months, 6.7% (*n* = 2) during 7–12 months, 23.3% (*n* = 7) during 13–24 months, and 26.7% (*n* = 8) for more than 24 months. One study (3.3%) did not specify the exact surveillance period. For AMR testing, 76.7% (*n* = 23) of studies performed MIC determination, which is recommended by WHO, with 33.3% (*n* = 10) and 53.3% (*n* = 16) using agar dilution and MIC gradient strip test, respectively, including 10.0% (*n* = 3) using both methods. Seven (23.3%) studies used only disc diffusion method for NG AMR testing, which is not recommended by WHO. Twenty (66.7%) studies reported using CLSI breakpoints, seven (23.3%) EUCAST breakpoints, and one (3.3%) both CLSI (for agar dilution results) and EUCAST (for MIC gradient strip test results) breakpoints. Two (6.7%) studies did not report the breakpoints used. The quality control of the AMR testing included the CLSI-recommended NG ATCC 49226 reference strain (*n* = 16, 53.3%), WHO NG reference strains (*n* = 12, 40.0%), other NG ATCC strains (*n* = 2, 6.7%), only clinical NG strains (*n* = 1, 3.3%), and five (16.7%) studies did not report any NG quality control strains (Table 1).

**Table 1:** *Neisseria gonorrhoeae* (NG) antimicrobial susceptibility/resistance studies in Africa from 2006 to 2020.^a^

References	Country	No. of isolates	Date of collection	Period (months)	Interpretative criteria	NG quality control strains	Test method	CRO R	CFM R	AZM R	CIP R
Maduna et al. (2020)	South Africa	27	March 2018 to April 2019	13	EUCAST	ATCC 49266 and ATCC 19424	DD	ND	ND	ND	ND
							AD	0%	0%	ND	ND
							MGS	0%	0%	15.0%	78.0%
Workneh et al. (2020)	Uganda	399	September 2016 to February 2018	17	CLSI	ATCC 49226	DD	1.0%	0%	ND	95.7%
							AD	ND	ND	ND	ND
							MGS	3.0%	0%	4.4%	100%
Nacht et al. (2020)	Kenya	35	January to July 2018	6	CLSI	UNK	DD	0%	ND	0%	34.0%
							AD	ND	ND	ND	ND
							MGS	ND	ND	ND	ND
Crucitti et al. (2020)^b^	Cameroon	449	2012 to 2018	84	EUCAST	UNK	DD	ND	ND	2.1%	ND
							AD	ND	ND	ND	ND
							MGS	1.8%	ND	ND	64.4%
Kakooza et al. (2021)	Uganda	458	March 2018 to September 2019	18	CLSI	ATCC 19424	DD	ND	ND	ND	ND
							AD	ND	ND	ND	ND
							MGS	0%	0%	0.2%	99.6%
Jacobsson et al. (2019)	South Africa	100	2015 to 2017	36	EUCAST	WHO A, F and P	DD	ND	ND	ND	ND
							AD	ND	ND	ND	ND
							MGS	0%	ND	0%	77.8%
Rambaran et al. (2019)	South Africa	319	September 2013 to Oct 2014	12	EUCAST	WHO F, K, L, O, and P	DD	ND	ND	ND	ND
							AD	0%	0.6%	26.6%	69.9%
							MGS	ND	ND	ND	ND
Mabonga et al. (2019)	Uganda	16	March to August 2015	5	CLSI	UNK	DD	33.3%	33.3%	ND	100%
							AD	0%	0%	0%	100%
							MGS	ND	ND	ND	ND
Yéo et al. (2019)	Cote d’Ivoire	212	January 2014 to December 2017	48	EUCAST	ATCC 49226 and 2008 WHO strains	DD	ND	ND	ND	ND
							AD	ND	ND	ND	ND
							MGS	0%	0%	6.1%	62.7%
Latif et al. (2018)	Zimbabwe	102	April 2015 to July 2016	15	CLSI	2008 WHO strains	DD	ND	ND	ND	ND
							AD	ND	ND	ND	ND
							MGS	0%	0%	10.0%	27.5%
Yeshanew and Geremew (2018)	Ethiopia	25	April 1 to August 30 2016	4	CLSI	ATCC 49226	DD	48.0%	ND	ND	52.0%
							AD	ND	ND	ND	ND
							MGS	ND	ND	ND	ND
Tayimetha and Unemo (2018)	Cameroon	193	2009 to 2014	69	CLSI	5 Clinical strains with different AMR phenotypes	DD	0%	ND	3.1%	17.6%
							AD	ND	ND	ND	ND
							MGS	ND	ND	ND	ND
Kularatne et al. (2018)	South Africa	128	2008 to 2017	120	CLSI: CIP, CRO, CFM EUCAST: AZM	2008 WHO strains	DD	ND	ND	ND	ND
							AD	ND	ND	ND	ND
							MGS	0%	0%	0%	69.0%
Mulu et al. (2017)	Ethiopia	13 (CIP), 8 (CRO)	2011 to 2014	48	CLSI	UNK	DD	35.8%	ND	ND	100%
							AD	ND	ND	ND	ND
							MGS	ND	ND	ND	ND
Ali et al. (2016)	Ethiopia	21	March to July 2015	5	CLSI	ATCC 49226	DD	0%	ND	ND	28.0%
							AD	ND	ND	ND	ND
							MGS	ND	ND	ND	ND
Duplessis et al. (2015)	Ghana	13	Jun 2012 to March 2013	10	UNK	ATCC 49226	DD	ND	ND	ND	ND
							AD	ND	ND	ND	ND
							MGS	0%	0%	ND	100%
Mulu et al. (2015)	Ethiopia	4	May to November 2013	6	CLSI	UNK	DD	ND	ND	ND	50.0%
							AD	ND	ND	ND	ND
							MGS	ND	ND	ND	ND
Takuva et al. (2014)	Zimbabwe	66	November 2010 to May 2011	6	CLSI	WHO F and K	DD	ND	ND	ND	ND
							AD	ND	ND	1.0%	ND
							MGS	0%	0%	ND	6.1%
Vandepitte et al. (2014)	Uganda	148	2008 to 2009	18	EUCAST	2008 WHO strains	DD	ND	ND	ND	ND
							AD	ND	ND	ND	ND
							MGS	0%	0.7%	16.2%	83.1%
Hailemariam et al. (2013)^c^	Ethiopia	11	1 December 2010 to 28 February 2011	3	CLSI	ATCC 49226	DD	27.8% (NS)	ND	ND	40.9% (NS)
							AD	ND	ND	ND	ND
							MGS	ND	ND	ND	ND
Hançali et al. (2013)	Morocco	72	July to December 2009	6	CLSI	ATCC 49226, WHO K and L	DD	ND	ND	ND	ND
							AD	ND	ND	ND	ND
							MGS	0%	0%	ND	86.8%
Lagace-Wiens et al. (2012)	Kenya	154	2009 and 2010	UNK	CLSI	ATCC 49226	DD	0%	0%	0%	53.2%
							AD	ND	ND	ND	ND
							MGS	0%	0%	0%	53.2%
Olsen et al. (2012)	Guinea-Bissau	31	February 2006 to January 2008	24	EUCAST	2008 WHO strains	DD	ND	ND	ND	ND
							AD	ND	ND	ND	ND
							MGS	0%	0%	0%	10.0%
Mehta et al. (2011)	Kenya	168	2002 to 2009	96	CLSI	ATCC 49226, WHO B, C and D	DD	ND	ND	ND	ND
							AD	0%	0%	0%	11%
							MGS	ND	ND	ND	ND
Brown et al. (2010)	Malawi	100	May to August 2007	3	CLSI	ATCC 49226	DD	ND	ND	ND	ND
							AD	0%	1.0%	ND	0%
							MGS	ND	ND	ND	ND
Apalata et al. (2009)	Mozambique	55	March to April 2005	1	CLSI	ATCC 49226	DD	ND	ND	ND	ND
							AD	0%	0%	ND	0%
							MGS	ND	ND	ND	ND
Cao et al. (2008)	CAM, CAR, MAD	CAM: 79, CAR: 30, MAD:126	March 2004 to June 2006	27	CLSI	ATCC 49226	DD	ND	ND	ND	ND
							AD	CAR: 0%, MAD: 0%	ND	ND	CAR: 0%, MAD: 2.9%
							MGS	ND	ND	ND	ND
Lewis et al. (2008)	South Africa	272	January to February 2007/January to April 2007	2 and 4	CLSI	WHO A-E, ATCC 49226	DD	ND	ND	ND	ND
							AD	ND	ND	ND	ND
							MGS	0%	ND	ND	30.0%
De Jongh et al. (2007)	South Africa	141	March 2004 to April 2005	13	UNK	WHO A-E, ATCC 49226	DD	ND	ND	ND	ND
							AD	0%	ND	ND	7%
							MGS	ND	ND	ND	7%
Moodley et al. (2006)^d^	South Africa	100	November 2003	1	CLSI	ATCC 49226	DD	ND	ND	ND	ND
							AD	0%	ND	ND	22.0%
							MGS	ND	ND	ND	ND

No., Number; NG, Neisseria gonorrhoeae; CRO, ceftriaxone; R, resistance; CFM, cefixime; AZM, azithromycin; CIP, ciprofloxacin; DD, disc diffusion; ND, Not determined (or complete); AD, agar dilution; MGS, MIC gradient strip test; UNK, Unknown; CAR, Central African Republic; CAM, Cameroon; MAD, Madagascar.

a There were no eligible studies in 2001–2005.

b Average antimicrobial resistance data from 2012 to 2018.

c Only non-susceptible (NS) results reported.

d Sample size in abstract and table differ (139 isolates and 100 isolates, respectively).

### *Neisseria gonorrhoeae* antimicrobial susceptibility/resistance in Africa, 2006–2020

The present review focused on studies examining susceptibility/resistance to the four main therapeutic antimicrobials, i.e., ceftriaxone, cefixime, azithromycin, and ciprofloxacin (Table 1). Thirteen (43.3%) studies examined all four of these antimicrobials using MIC determination, which is recommended by WHO. Studies performing ciprofloxacin MIC determination (n = 23) reported high levels of resistance, i.e., 15 (65.2%) studies reported ≥ 30% ciprofloxacin resistance and four (17.4%) studies (from Ghana and Uganda) reported > 99% ciprofloxacin resistance. Fourteen (46.7%) studies performed MIC determination for azithromycin and azithromycin resistance ranged from 0% to 26.6%, with five (35.7%) studies (from Cote d’Ivoire, South Africa, Uganda, and Zimbabwe) reporting > 5% azithromycin resistance (6.1%–26.6%). Twenty-three (76.7%) studies and 17 (56.7%) studies used MIC determination for ceftriaxone and cefixime, respectively. Two (8.7%) studies (from Cameroon and Uganda) reported low levels of ceftriaxone resistance (1.8% and 3.0%, respectively) and three (17.6%) studies (from Malawi, South Africa and Uganda) identified low levels of cefixime resistance (0.6%–1%; Table 1).

## Discussion

We report an exceedingly low level of NG AMR surveillance in Africa with the use of mixed and frequently suboptimal approaches, protocols and methodologies for antimicrobial susceptibility testing, sample size determination and quality assurance, which make inter-study and inter-country comparisons of NG AMR situations difficult. It is also a major concern that most AMR surveillance was performed as *ad hoc* research studies and not in routine standardized and quality-assured NG AMR surveillance programs. Additional main concerns are that many studies examined a suboptimal number of isolates, reporting less than the 100 isolates per year and setting that are recommended by the WHO GASP, EGASP, and GLASS (Wi et al., 2017; Unemo et al., 2019a,b, 2021), the representativeness of the examined isolates was unclear in many of the studies, and very limited patient metadata (clinical, demographical and epidemiological) were reported. It is imperative to urgently establish an enhanced, standardized and quality-assured NG AMR surveillance in Africa, the region with the highest gonorrhea incidence globally.

The management and control of gonorrhea is dependent on the availability of effective, affordable and accessible antimicrobial treatment (Unemo, 2015; World Health Organization, 2016; Wi et al., 2017; Unemo et al., 2021). WHO empiric global treatment guidelines recommend the use of ceftriaxone/cefixime in combination with azithromycin as single-dose therapy, i.e., in settings where there is no local AMR data (World Health Organization, 2016). The recommendation of dual therapy is intended to cure also all ceftriaxone/cefixime-resistant gonorrhea cases, i.e., to avoid their further transmission, as well as to cure additional STIs, such as *Chlamydia trachomatis* infections, when used in syndromic management. However, a high-dose ceftriaxone monotherapy, which has been recently introduced in some regions and countries (Fifer et al., 2020; St Cyr et al., 2020; Unemo et al., 2020), is currently considered also for the WHO global gonorrhea treatment guideline. Nevertheless, for this type of recommendation improved NG AMR surveillance data for relevant therapeutic antimicrobials in Africa and many additional global settings are imperative. The WHO GASP, EGASP and GLASS (Wi et al., 2017; Unemo et al., 2019a, 2021) recommend mandatorily testing (i.e., where antimicrobial discs, MIC gradient strips or antimicrobials for agar dilution are available) of NG susceptibility to ceftriaxone, cefixime, and azithromycin, which are gonorrhea first-line or second-line antimicrobials in most international treatment guidelines (World Health Organization, 2016; Fifer et al., 2020; St Cyr et al., 2020; Unemo et al., 2020). However, in the present study it was found that many of the publications from the African continent were not eligible for inclusion because they examined antimicrobials excluded from the international gonorrhea treatment guidelines since decades and for which the resistance levels are high, such as penicillins, tetracycline and ciprofloxacin, which may be associated with availability of these antimicrobials (Table 1). The primary focus on any NG AMR surveillance should be to examine susceptibility to antimicrobials currently recommended for treatment in evidence-based international guidelines as well as in national guidelines. However, it was a grave concern to see that ciprofloxacin remained recommended and/or used for treatment of gonorrhea in many African countries and, based on the very high levels of ciprofloxacin resistance in Africa as well as globally (Table 1; Unemo et al., 2021), ciprofloxacin should not be continuously recommended or used for gonorrhea treatment.

Notably, in the latest WHO GASP/GLASS publication reporting NG AMR results from 2017 to 2018 (Unemo et al., 2021), only 11% (5/47) of the countries in the WHO African Region reported data on susceptibility/resistance to ceftriaxone and azithromycin. Madagascar (1.2%, 1/81) and Uganda (0.3%, 1/340) reported occasional isolates with resistance or decreased susceptibility to ceftriaxone and Kenya reported azithromycin-resistant isolates (5.3%, 5/96; Unemo et al., 2021). In the present review, two eligible included studies reported low levels of ceftriaxone resistance using MIC determination (1.8% in Cameroon and 3.0% in Uganda). Furthermore, five studies from four countries reported more than 5% azithromycin resistance (WHO’s threshold for considering exclusion of an antimicrobial in the recommended gonorrhea treatment) using MIC determination (6.1–26.6%; in Cote d’Ivoire, South Africa, Uganda, and Zimbabwe; Table 1). Seven (23.3%) studies used only qualitative disc diffusion method and three of these studies reported exceedingly high levels of resistance to ceftriaxone (35.8%–69.0%, Table 1). However, when two of these studies (35.8% and 48.0% ceftriaxone resistance) did not subsequently verify their disc diffusion results using MIC determination and one study (69.0% ceftriaxone resistance) reported 0% ceftriaxone resistance using MIC determination, the ceftriaxone resistance levels using disc diffusion were considered unrealistic and inaccurate. Unfortunately, disc diffusion methods for antimicrobial susceptibility testing in NG have suboptimal correlation with MIC determination methods such as agar dilution and MIC gradient strip test. If disc diffusion methods are used, e.g., due to limited resources or laboratory capacity, it is imperative with a high level of quality assurance and that rare resistance, e.g., to ceftriaxone, is subsequently verified using MIC determination. Anyway, occasional NG strains with resistance or decreased susceptibility to ceftriaxone or azithromycin are evidently spreading also in the WHO African Region.

Consequently, it is essential to substantially enhance, standardize and quality-assure the NG AMR surveillance in the WHO African region. However, many obstacles need to be overcome to achieve this aim. National and international leadership, political (e.g., at national Ministries of Health) and financial commitments are imperative. However, it is also important to increase the awareness among healthcare staff (at clinics, laboratories and public health organizations) that regular, representative and quality-assured NG AMR surveillance should be the foundation of national AMR action plans to manage and control gonorrhea, part of routine diagnostics and/or surveillance, and used to inform revisions of national gonorrhea treatment recommendations. Due to the fastidious nature of NG, it is additionally essential to provide training to staff at clinics and laboratories concerning appropriate: (1) sample collection, transportation and preservation; (2) laboratory methodologies, especially high-quality sensitive and specific culture and AMR testing; and (3) quality assurance, including use of internal quality controls and external quality assessment (Wi et al., 2017; Unemo et al., 2019a). Finally, to increase the recruitment of gonorrhea patients it is important to mitigate the over-the-counter availability of antimicrobials, i.e., without prescription from medical doctor, and decrease stigmas associated with STIs that both negatively impact the number of men and women that attend medical care when have symptoms of an STI or other suspicion of being infected with an STI. To support the enhancement of the NG AMR surveillance in the WHO African region, it is recommended that the WHO GASP and especially WHO EGASP are further expanded in this region. The WHO EGASP uses optimized, standardized and quality assured protocols for selection and size of sample (surveillance population), clinical management of patient, microbiological procedures, collection of patient metadata (clinical, microbiological, demographical and epidemiological), quality assurance of all procedures and reporting. Briefly, the WHO EGASP protocols recommend a sample size of at a minimum 100 representative gonorrhea patients and accordingly NG isolates per year and setting, to use MIC determination of at a minimum ceftriaxone, cefixime and azithromycin (agar dilution or MIC gradient strip tests, i.e., Etests) and recommended interpretative breakpoints, and to integrate internal and external quality assurance in all procedures (e.g., using WHO reference strains (Unemo et al., 2016) to quality assure the laboratory component). All these standardized protocols, procedures and components of quality assurance enable comparability internationally in WHO EGASP. WHO EGASP is currently expanded as well as further developed, i.e., to include test of cure and genome sequencing, where feasible. Regular quality-assured genome sequencing in conjunction with AMR and patient metadata has been shown to be ideal for public health surveillance including AMR surveillance and crucial to explain fluctuations in gonorrhea epidemiology, circulating AMR and antimicrobial-susceptible NG clones, lineages and their associations with patient groups, nationally and internationally (Harris et al., 2018; Sánchez-Busó et al., 2022).

In conclusion, it is imperative to establish an enhanced, standardized and quality-assured NG AMR surveillance, ideally including patient metadata and genome sequencing as in WHO EGASP, in Africa, which is the region with the highest gonorrhea incidence globally. This will enable the monitoring of AMR trends, detection of emerging AMR, and timely refinements of national and international gonorrhea treatment guidelines. To achieve this aim, national and international leadership, political and financial commitments are imperative.

## Author contributions

FK, RK, and MU conceived and designed the study and wrote the first draft of the manuscript. GM, JK, ON, PK, BS, and DG supported the literature review. All authors contributed to the article and approved the submitted version.

## Conflict of interest

The authors declare that the research was conducted in the absence of any commercial or financial relationships that could be construed as a potential conflict of interest.

## Publisher’s note

All claims expressed in this article are solely those of the authors and do not necessarily represent those of their affiliated organizations, or those of the publisher, the editors and the reviewers. Any product that may be evaluated in this article, or claim that may be made by its manufacturer, is not guaranteed or endorsed by the publisher.
